# Effect of Chromatin-Remodeling Agents in Hepatic Differentiation of Rat Bone Marrow-Derived Mesenchymal Stem Cells* In Vitro* and* In Vivo*

**DOI:** 10.1155/2016/3038764

**Published:** 2016-05-08

**Authors:** Danna Ye, Tong Li, Philip Heraud, Rangsun Parnpai

**Affiliations:** ^1^Embryo Technology and Stem Cell Research Center, School of Biotechnology, Suranaree University of Technology, 111 University Avenue, Muang District, Nakhon Ratchasima 30000, Thailand; ^2^Reproductive Medicine Center, The First Affiliated Hospital of Wenzhou Medical University, Wenzhou, Zhejiang 325000, China; ^3^School of Ophthalmology and Optometry, Eye Hospital, Wenzhou Medical University, Wenzhou, Zhejiang 325000, China; ^4^Department of Anatomy and Developmental Biology, Faculty of Medicine, Nursing & Health Sciences, Monash University, Clayton, VIC 3800, Australia; ^5^Centre for Biospectroscopy, School of Chemistry, Monash University, Clayton, VIC 3800, Australia

## Abstract

Epigenetic events, including covalent histone modifications and DNA methylation, play fundamental roles in the determination of lineage-specific gene expression and cell fates. The aim of this study was to determine whether the DNA methyltransferase inhibitor (DNMTi) 5-aza-2′-deoxycytidine (5-aza-dC) and the histone deacetylase inhibitor (HDACi) trichostatin A (TSA) promote the hepatic differentiation of rat bone marrow-derived mesenchymal stem cells (rBM-MSCs) and their therapeutic effect on liver damage. 1 *μ*M TSA and 20 *μ*M 5-aza-dC were added to standard hepatogenic medium especially at differentiation and maturation steps and their potential function on hepatic differentiation* in vitro* and* in vivo* was determined. Exposure of rBM-MSCs to 1 *μ*M TSA at both the differentiation and maturation steps considerably improved hepatic differentiation. TSA enhanced the development of the hepatocyte shape, promoted the chronological expression of hepatocyte-specific markers, and improved hepatic functions. In contrast, treatment of rBM-MSCs with 20 *μ*M 5-aza-dC alone or in combination with TSA was ineffective in improving hepatic differentiation* in vitro*. TSA and/or 5-aza-dC derived hepatocytes-like cells failed to improve the therapeutic potential in liver damage. We conclude that HDACis enhance hepatic differentiation in a time-dependent manner, while DNMTis do not induce the hepatic differentiation of rBM-MSCs* in vitro*. Their* in vivo* function needs further investigation.

## 1. Background

Liver development from the endodermal layer is known to proceed via several distinct steps that involve extracellular signals induced by growth factors and cytokines [1]. Numerous cytokines and growth factors have been shown to have potent effects on hepatic growth and differentiation* in vitro* [2–4]. The importance of the sequential addition of liver-specific factors in a time-dependent manner that resembles the secretion pattern during liver embryogenesis has been demonstrated [3]. A variety of biochemical cocktails have been developed for promoting the differentiation of adult stem cells into hepatocytes [2–5]. However, the potential of differentiation attained using existing methods remains low. The mechanisms through which mesenchymal stem cells (MSCs) overcome lineage borders and transdifferentiate to hepatocytes are unclear. Initial attempts at improving differentiation methods focused on mimicking* in vivo* conditions and on the addition of soluble medium components. Recently, epigenetic modifications during differentiation have received much research attention, due to their fundamental role in controlling differentiation [6]. Epigenetic modifiers, including DNA methyltransferase inhibitors (DNMTis), such as 5-aza-2′-deoxycytidine (5-aza-dC) and 5-azacytidine, and histone deacetylase inhibitors (HDACis), such as trichostatin A (TSA) and dimethyl sulfoxide, are commonly used.

TSA is an organic compound that specifically inhibits class I and class II mammalian histone deacetylases (HDACs) by directly binding to the catalytic site of HDAC [7]. TSA interferes with the removal of acetyl groups from histones (i.e., the function of HDACs) and thereby alters the ability of DNA transcription factors to access the DNA molecules inside chromatin [8]. Histone acetylation is generally associated with gene activation. Studies have shown that, after exposure to TSA, the phenotype of normal primary rat hepatocytes was maintained in* in vitro* cultures, implying that epigenetic alterations could represent a method to develop phenotypically stable primary hepatocyte cultures [9, 10]. Chromatin remodeling plays a central role in the regulation differentiation and stem cell functions during organogenesis. Studies have demonstrated that when cultured human bone marrow-derived MSCs (BM-MSCs) and rat mesenchymal progenitor cells pretreated for 6 days with hepatogenic stimulating agents were exposed to 1 *μ*M TSA, functional hepatocytes were obtained. This indicates that TSA can function as a stimulant during or after hepatic differentiation [11, 12].

5-Aza-dC is a strong inducer of DNA demethylation. It is an analogue of cytosine, which when incorporated into DNA irreversibly binds methyltransferase enzymes as they attempt to methylate cytosine analogues. This depletion of methyltransferases in the cell results in passive demethylation, which is known to reactivate epigenetically silenced genes [13]. 5-Aza-dC has been used to maintain differentiation in normal mouse primary hepatocytes [14]. Exposure to 5-aza-dC for 24 h prior to hepatic stimulation successfully induced hepatic differentiation of murine BM-MSCs [15], rat adipose tissue-derived stem cells [16], human BM-MSCs [17], and human umbilical cord blood MSCs [18]. The above results showed that 5-aza-dC can function as a preconditioning agent prior to hepatic differentiation [6].

Until now, HDACis and DNMTis have usually been applied separately, and no study has compared the effects of combined and single exposures to 5-aza-dC and TSA on the process of hepatogenesis* in vitro* and* in vivo*. We aimed to determine and compare the effects of single and combined exposure to these chromatin-remodeling agents during hepatogenesis on the differentiation of rBM-MSCs to hepatocytes* in vitro* and their therapeutic potential in liver damage.

## 2. Materials and Methods

### 2.1. Hepatic Differentiation

All animal care procedures and surgical interventions were undertaken in strict accordance with the approval of the Laboratory Animals Ethics Committee of Suranaree University of Technology. We isolated rBM-MSCs from 8-week-old female Wistar rats and cultured them as previously described [1, 19]. The standard hepatogenic protocol was used [1, 19]. In brief, rBM-MSCs at passage five were serum-deprived for 2 days (conditioning step) in Iscove's Modified Dulbecco's Medium (IMDM) supplemented with 10 ng/mL basic fibroblast growth factor (bFGF) and 20 ng/mL epidermal growth factor (EGF). We followed a 2-step protocol. In step 1 (differentiation step), IMDM supplemented with 20 ng/mL hepatocyte growth factor (HGF), 10 ng/mL bFGF, and 4.9 mmol/mL nicotinamide was applied to the rBM-MSCs for 7 days. In step 2 (maturation step), the cells were treated with IMDM supplemented with 10 mmol/mL ITS (insulin, transferrin, and selenious acid), 1 mmol/mL dexamethasone, and 20 ng/mL oncostatin M for 14 days. The media were changed twice weekly. Different chromatin-remodeling agents were added to the standard hepatogenic medium at different time points. The culture conditions (Table 1) were as follows. (1) Group 1 (G1): rBM-MSCs were pretreated with 20 *μ*M 5-aza-dC for 24 h, and 1 *μ*M TSA was added during both the differentiation and maturation steps. (2) Group 2 (G2): rBM-MSCs were pretreated with 20 *μ*M 5-aza-dC for 24 h, and 1 *μ*M TSA was added during the maturation step. (3) Group 3 (G3): rBM-MSCs were pretreated with 20 *μ*M 5-aza-dC for 24 h. (4) Group 4 (G4): 1 *μ*M TSA was added during both the differentiation and maturation steps. (5) Group 5 (G5): 1 *μ*M TSA was added during the maturation step. (6) Group 6 (G6): the standard hepatogenic medium was used (control group). All reagents were purchased from Sigma Aldrich, unless otherwise indicated.

### 2.2. Characterization of Hepatocyte-Like Cells

Assays for the expression of liver-specific proteins and genes and to determine liver function were conducted using our previously described protocol [1, 19].

### 2.3. Cell Transplantation

Liver damage was induced by dimethylnitrosamine (DMN) in rats. After being detached from the plate by trypsin/EDTA treatment, the differentiated cells from each experimental group were suspended in L mL phosphate buffer saline for each donor aliquot at a concentration of 1 × 10^6^ cells/mL. The DMN treated rats were randomly divided into seven groups after 4 weeks of DMN treatment and injected differentiated cells. DMN untreated rats were regarded as normal group. DMN treated rats without injecting differentiated cells were regarded as control group. On day 28, venous blood was collected and all rats were killed, and liver tissues were harvested for analysis.

### 2.4. Histopathology of the Liver

Frozen liver samples (approx. 0.5 cm^3^) were randomly taken from the right, median, and left lobes of each rat liver and embedded in optimal temperature cutting (OCT, Sakura Finetek USA, Torrance, CA) compound and sectioned consecutively at 10 *μ*m in a cryostat at −18°C (Leica Biosystems, Nussloch, Germany). For Hematoxylin and Eosin staining (H&E), the liver sections were mounted on slides and air dried for at least 20 minutes followed by fixation in 10% formalin for 30 seconds. Then these sections were stained with routine H&E according to regular staining procedure such as hydration, staining, dehydration, and clearing. The stained slides were finally covered with a cover-slip using Entellen mounting medium (Electron Microscopy Science, Hatfield, PA, USA).

### 2.5. Assessment of Liver Function

Blood samples were obtained from each rat and centrifuged for 30 minutes at 600 ×g and serum collected. Albumin (ALB), aspartate aminotransferase (AST), and alanine transaminase (ALT) levels were assessed using conventional laboratory methods [19].

### 2.6. Statistics

The number of cells positive for a given marker was determined by counting the number of cells positive for that marker at least 4 fields and among a total of 1000 cells. All values are presented as mean ± SEM, and the data were performed for statistical significance using one-way ANOVA with *p* < 0.05 considered statistically significant.

## 3. Results

### 3.1. Morphological Features

**Figure 1:**
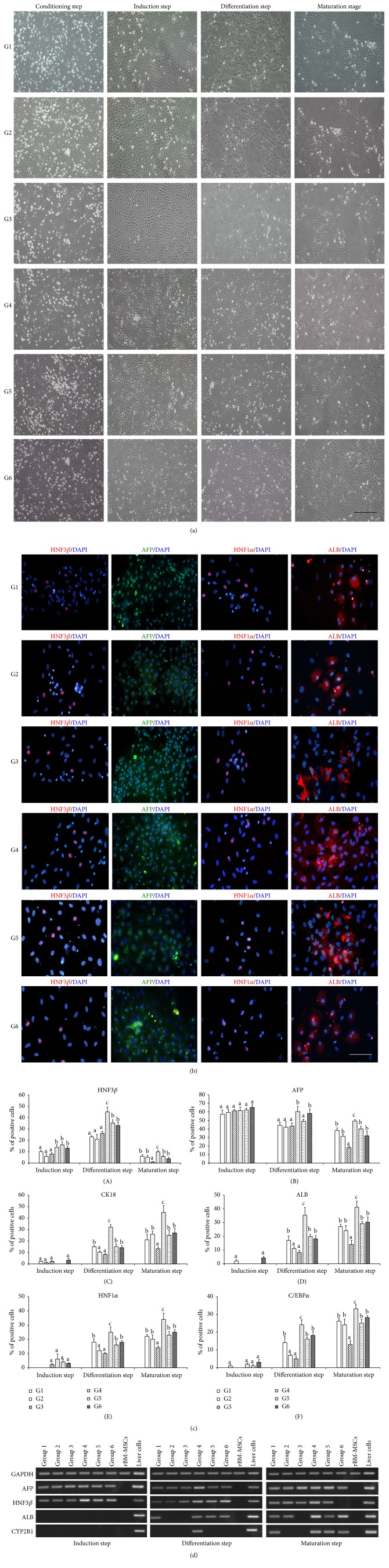
Characterization of differentiated cells. (a) Changes in cell morphology during the hepatic differentiation of rBM-MSCs. Scale bar: 500 *μ*m. (b) The expression of HNF3*β*, AFP, HNF1*α*, and ALB on the last day of hepatic differentiation in each experimental group, assessed using immunocytochemistry. Scale bar: 200 *μ*m. (c) Expression of hepatocyte-specific proteins after cell exposure to chromatin-remodeling agents. Immunocytochemistry was performed for HNF3*β*, AFP, CK18, ALB, HNF1*α*, and C/EBP*α*. Values represent means ± SEM. Bars with different superscripts in the certain step are different statistically (*p* < 0.05). ^a, b^ *p* < 0.05, ^b, c^ *p* < 0.05, and ^a, c^ *p* < 0.01. (d) RT-PCR analyses of the temporal expression patterns of selected hepatocyte-specific genes during the hepatic differentiation of rBM-MSCs.

We found that 5-aza-dC did not affect the cell morphology in the treatment groups (G1, G2, and G3) during the pretreatment and conditioning steps. The cells in these groups presented a fibroblastic shape (Figure 1(a)). In the induction step, the cell morphology in all experimental groups developed to an epithelioid shape. The cells in the 5-aza-dC-treated groups (G1, G2, and G3) exhibited a 3-day delay in showing these morphological changes, as compared to the cells in the control group (G6). As the differentiation progressed, the change in cellular morphology was gradual in all experimental groups. In the differentiation step, islands of adherent round or polygonal cells surrounded by spindle-shaped cells were observed in all experimental groups. During this step, remarkable changes in cell morphology were observed in G4 (TSA treatment during differentiation and maturation); the cells in this group displayed a hepatocyte-like morphology, characterized by cytoplasmic granulation and a central nucleus with prominent nucleolus. This morphology was not observed in the control group (G6), which indicates that TSA promoted hepatic differentiation. In the maturation step, the cells underwent drastic morphological changes in all experimental groups, as compared to the morphology at the beginning of differentiation. However, the size of the cell islands differed among the groups. The largest islands were seen in G4 (TSA exposure during differentiation and maturation), while the smallest islands were seen in G3 (5-aza-dC pretreatment only). The cell islands in G1, G2, G5, and G6 were of the same size.

### 3.2. Liver-Specific Protein Expression

To determine whether the morphological changes in the cells treated with 5-aza-dC and/or TSA were associated with changes in the expression patterns of proteins, we conducted immunocytochemical tests to determine the expressions of early (hepatocyte nuclear factor [HNF]3*β*, *α*-fetoprotein [AFP]), mid-to-late (albumin [ALB], cytokeratin 18 [CK18]), and late (HNF1*α*, CCAAT/enhancer-binding protein-*α* [C/EBP*α*]) hepatic differentiation markers. Cells from all experimental groups chronologically expressed HNF3*β*, AFP, ALB, CK18, HNF1*α*, and C/EBP*α* (Figure 1(b)), but the patterns and levels of expression were different in each group (Figure 1(c)). The positivity rate was calculated by counting the number of positive cells out of a total of 1000 cells in each experimental group. In the induction step, the number of HNF3*β*-positive cells in the 5-aza-dC-treated groups (G1, 10%  ±  0.78%; G2, 6%  ±  1.85%; and G3, 8%  ±  1.03%) was significantly lower than that in the control group (G6, 13%  ±  1.38%) (^a, b^ *p* < 0.05), but the number of AFP-positive cells was not significantly different. This is consistent with the morphological changes observed and indicates that 5-aza-dC delays hepatic differentiation. Expression or low expression of ALB, CK18, HNF1*α*, and C/EBP*α* was not observed in any of the experimental groups. In the differentiation step, the number of positive cells increased for all analyzed markers, except AFP. In cells treated with TSA only (G4), the number of HNF3*β*-, CK18-, ALB-, HNF1*α*-, and C/EBP*α*-positive cells was significantly higher than that in the control group (G6; 45%  ±  4.23% versus 33%  ±  3.07%; 32%  ±  2.35% versus 14%  ±  1.71%; 35%  ±  5.83% versus 18%  ±  2.66%; 25%  ±  3.66% versus 18%  ±  1.02%; 24%  ±  2.74% versus 18%  ±  2.14%, resp.) (^b, c^ *p* < 0.05). The number of positive cells for all analyzed markers was significantly lower in the groups with 5-aza-dC treatment only (G2, G3) than in the control group (G6). There were no significant differences in the number of cells positive for ALB (17%  ±  3.1% versus 18%  ±  2.66%), CK18 (15%  ±  0.78% versus 14%  ±  1.71%), HNF1*α* (18%  ±  2.33% versus 18%  ±  1.02%), and C/EBP*α* (14%  ±  3.5% versus 18%  ±  2.14%) between the group that was treated with a combination of TSA and 5-aza-dC (G1) and the control group (G6). In the maturation step, the cells in the control group (G6) expressed AFP, ALB, CK18, HNF1*α*, and C/EBP*α* and low levels of HNF3*β*, indicating complete hepatocyte differentiation. The number of cells that expressed HNF3*β*, AFP, ALB, CK18, HNF1*α*, and C/EBP*α* was significantly higher in G4 than in the control group (G6; 10%  ±  0.99% versus 4%  ±  0.86%; 49%  ±  1.85% versus 32%  ±  4.86%; 41%  ±  4.29% versus 30%  ±  3.85%; 45%  ±  6.02% versus 27%  ±  3.96%; 34%  ±  4.39% versus 25%  ±  1.68%; 33%  ±  2.39% versus 28%  ±  1.08%, resp.) (^b, c^ *p* < 0.05). The number of positive cells for all analyzed markers in G1, G2, and G5 did not significantly differ from that in the control group (G6). The group with 5-aza-dC treatment only (G3), however, was found to have lower expression of all analyzed markers than that in the control group (G6).

### 3.3. Liver-Specific Gene Expression

To determine whether the morphological changes observed were sustained and associated with the induction of hepatocyte-specific genes, total RNA was isolated during the induction, differentiation, and maturation steps. Reverse transcription polymerase chain reaction was used to analyze the expression of early (HNF3*β* and AFP) and mid-to-late (ALB and CYP2B1) gene markers of hepatic differentiation (Figure 1(d)). Undifferentiated rBM-MSCs and rat adult liver cells were used as negative and positive controls, respectively. In the induction step, the cells in all experimental groups expressed HNF3*β* and AFP, but not ALB and CYP2B1. In the differentiation step, ALB expression was detected in G6 (control), G1, G4, and G5, but not in G2 and G3. In addition, CYP2B1 expression was found in only G4. HNF3*β* and AFP were continued to be expressed in all experimental groups. In the maturation step, the cells in the control group (G6) lost their HNF3*β* expression and began to express CYP2B1. The expression patterns of all genes observed in G1, G4, and G5 were the same as those in adult liver cells. Moreover, the cells in all experimental groups continued to express AFP, and ALB expression was detected in all experimental groups.

### 3.4. Hepatic Function

**Figure 2:**
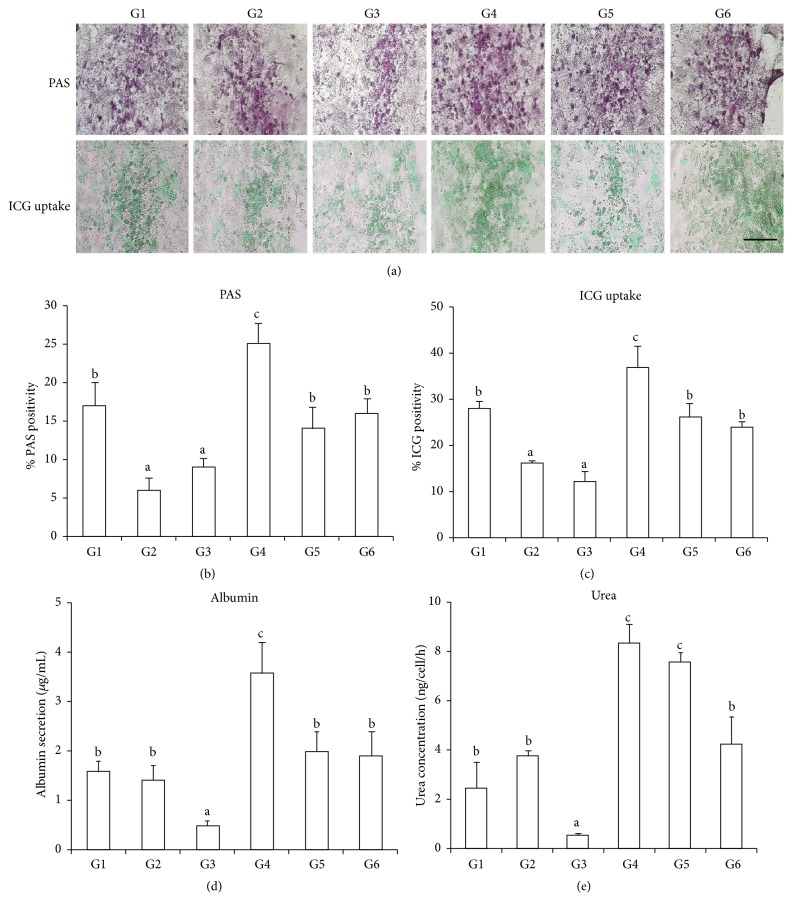
Comparative analysis of hepatocyte-like functions on the last day of hepatic differentiation. (a) Periodic acid Schiff (PAS) assays showing glycogen-positive cells and ICG uptake in each experimental group. (b and c) Number of glycogen-positive cells. (d) ALB secretion. (e) Urea production. Bars with different superscripts are different statistically (*p* < 0.05). ^a, b^ *p* < 0.05, ^b, c^ *p* < 0.05. Scale bar: 100 *μ*m.

To evaluate whether the rBM-MSC-derived hepatocyte-like cells also acquired typical hepatic functions, we analyzed ALB and urea secretion, glycogen production and storage, and indocyanine green (ICG) uptake on the last day of hepatic differentiation (Figure 2). Cells exposed to TSA during both differentiation and maturation (G4) exhibited significantly increased glycogen production, ICG uptake, and ALB and urea secretion when compared with the control group (G6) cells. The lowest levels of these functions were found in the cells treated with 5-aza-dC only (G3), which is consistent with the previous result that TSA promotes hepatic differentiation and 5-aza-dC does not improve hepatic differentiation potential.

### 3.5. Cell Transplantation

**Figure 3:**
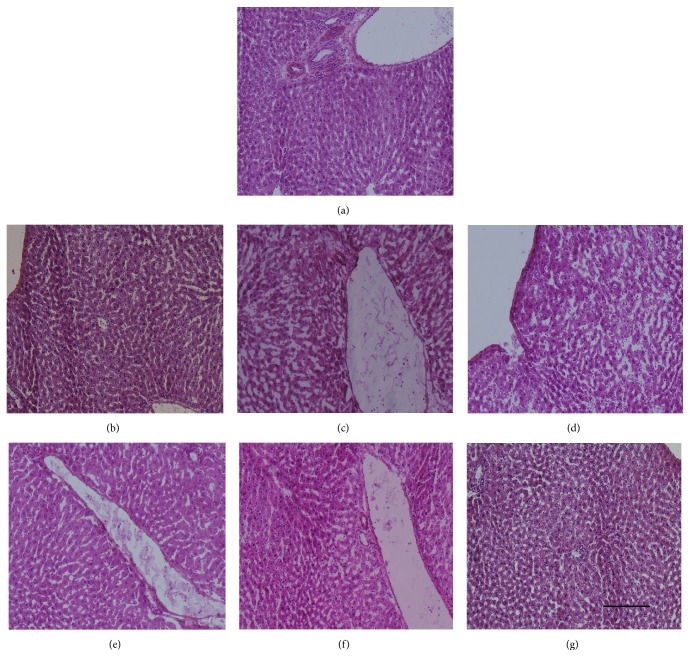
Hematoxylin and Eosin staining of liver sections. (a) Normal liver; (b–g) G1 to G6. Original magnification, 100x.

To evaluate whether these chromatin-remodeling-derived hepatocyte-like cells also had therapeutic effect on liver damage* in vivo*, we transplanted those cells to liver damage rats' model. The effects of chromatin-remodeling-derived hepatocyte-like cells on DMN-injured liver were evaluated by histopathologic examination of the liver sections by H&E staining (Figure 3). The G2 and G3 (Figures 3(c) and 3(d)) exhibited the hemorrhagic necrosis and disruption of tissue architecture compared to normal liver (Figure 3(a)). Hemorrhagic necrosis was rarely observed in the G1, G4, G5, and G6 (Figures 3(b), 3(e), 3(f), and 3(g)) and showed to be similar to normal liver (Figure 3(a)).

**Figure 4:**
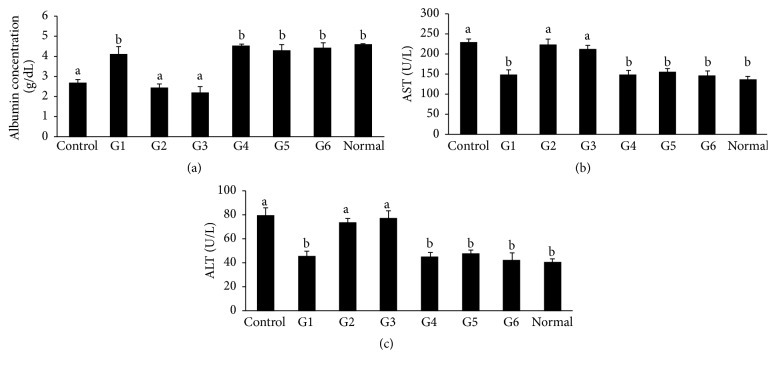
Biochemical analysis of blood sera. (a) Concentration of albumin (ALB), (b) aspartate aminotransferase, and (c) alanine transaminase (ALT) in blood serum of each experimental group. Bars with different superscripts are different statistically (*p* < 0.05). ^a, b^ *p* < 0.05, ^b, c^ *p* < 0.05. DMN-injured rats were considered as control group.

We also detected serum levels of albumin (ALB), aspartate aminotransferase [3], and alanine transaminase (ALT). As shown in Figure 4(a), the serum ALB levels in the G1, G4, and G5 were significantly higher than control level indicating the transplanted cells restored albumin production but these were still at the same level compared to G6 which did not add any chromatin-remodeling agent. In contrast, the serum ALB levels in the G2 and G3 were close to the control level. The G1, G4, and G5 were shown to significantly suppress the serum AST and ALT levels to the normal level indicating the transplanted cells suppression of inflammation; however these were still not significant compared to G6 (Figures 4(b) and 4(c)). The serum levels of AST and ALT in G2 and G3 were significantly higher than normal level and close to control level.

## 4. Discussion

The objective of this study was to determine the effects of 5-aza-dC and TSA, separately and in combination, on the differentiation of rBM-MSCs to hepatocytes* in vitro* and* in vivo*. Our results showed that 1 *μ*M TSA enhanced hepatic differentiation when it was added at both the differentiation and maturation steps (G4). Specifically, TSA induced early and obvious differentiation to hepatocyte-like cells, caused a prolonged and stable increase in the overall expression levels of typical hepatic proteins, and enhanced hepatic functions (albumin and urea secretion, glycogen production, and ICG uptake). However, these changes depended on the timing of TSA exposure. When cells were exposed to TSA in the maturation step (G5), neither hepatic maturation nor hepatic function was improved. Our results are consistent with those reported by Snykers et al. [11]: the addition of TSA to cultured human BM-MSCs pretreated for 6 days with hepatogenic stimulating agents triggered their transdifferentiation into cells with similar phenotypic and functional characteristics as primary hepatocytes. Similar results have been obtained with rat bone marrow-derived mesenchymal progenitor cells, which when exposed to TSA from day 6 of hepatic differentiation onwards exhibited significantly improved hepatic differentiation [12]. TSA seems to have the potential to overcome cell fate determinism, cross lineage borders, and favor lineage-specific differentiation [6]. Stimulation with TSA failed to promote oligodendrocyte differentiation in rat neural progenitor cells but could trigger neural cell differentiation under neural stimulating conditions; this indicated that the timing of exposure is an important factor affecting TSA function [20]. Our results showed that exposure to TSA during only the maturation step failed to promote hepatic differentiation, possibly because the timing of exposure was incorrect. Although mechanistic insights into how TSA regulates the transcription of lineage-specific genes are at present largely unresolved, other studies and our own results have demonstrated that prestimulation of cells towards the intended selected direction prior to the introduction of TSA may be a key determinant of the crossing of lineage boundaries and promotion of transdifferentiation into a specific lineage by means of TSA exposure.

We also found that exposure to 5-aza-dC only did not improve hepatic differentiation potential (G3), as evidenced by the low hepatic maturation and function. This result contradicts the previously reported finding that 5-aza-dC functions as a preconditioning agent prior to hepatic differentiation [15–18, 21]. In addition, TSA more or less compensated for 5-aza-dC treatment (G1 and G2) in a time-independent manner, as indicated by the improvement in hepatic maturation and function. This indicated the synergetic or synergistic behavior of 5-aza-dC and TSA in respect to hepatic differentiation processes [14]. The reasons for these controversial results are unknown but might be related to differences in the sources of MSCs and in microenvironments. Successful cell fate manipulation highly relies on the cell microenvironment (cell-cell contact, cell densities), the appropriate type of epigenetic modifier, and the optimal fine-tuning of its dose and timing (onset and duration) of exposure [12, 22–24]. The suitability of HDACis and/or DNMTis to promote hepatic transdifferentiation requires a delicate balance between proliferation and differentiation, between biological activity/pharmacokinetic properties and toxicological characteristics, and finally between apoptosis and cell survival [24]. In at least some cases, the failure of lineage-specific differentiation could be ascribed to the inaccurate timing of exposure to and dosage of chromatin-modulating agents. Basically, although not generally, prestimulation of cells towards the intended selected direction prior to the introduction of HDACis may be a key determinant of the crossing of lineage boundaries and promotion of transdifferentiation into a specific lineage by means of HDAC inhibition [11, 12, 22, 25–29].

Our latest results indicated that transplantation of rBM-MSCs derived hepatocyte-like cells effectively treats liver disease in rat (unpublished data). In this study, we aimed to study whether transplantation of TSA and/or 5-aza-dC derived hepatocyte-like cells had therapeutic potential on liver disease based on our current report. Our results showed that transplantation of 5-aza-dC alone or in combination with TSA at the maturation stage derived hepatocyte-like cells failed to treat liver disease, consistent with the fact that 5-aza-dC was ineffective in improving hepatic differentiation* in vitro*. The lower number of transplanted hepatocyte-like cells led to inefficiency of treatment of liver damage. Transplantation of TSA alone or in combination with 5-aza-dC at the differentiation and maturation stage derived hepatocyte-like cells suppressed liver fibrosis; however, the efficiency was not significantly improved compared to the control. The possible reason may be because of the heterogeneous population of TSA alone or in combination with 5-aza-dC at the differentiation and maturation stage derived hepatocyte-like cells. Our results showed that TSA alone or in combination with 5-aza-dC at the differentiation and maturation stage increased and prolonged expression of the immature makers HNF3*β* and AFP. Normally, AFP expression is known to drop down along the progression of hepatic maturation [30]. The continued AFP expression in TSA alone or in combination of 5-aza-dC at the differentiation and maturation stage derived hepatocyte-like cells may indicate the cells have different degrees of maturation phenotype. Additional investigation is needed to purify hepatocyte-like cells before transplantation.

## 5. Conclusions

TSA enhances the hepatic differentiation of rBM-MSCs* in vitro*. TSA caused early and obvious induction of hepatocyte-like cells, produced a prolonged and stable increase in the overall expression levels of typical hepatic proteins, and enhanced hepatic function, in a time-dependent manner. In contrast, exposure to 5-aza-dC, either alone or in combination with TSA, did not improve hepatic differentiation* in vitro*. Yet, further* in vivo* investigation is needed.

## Figures and Tables

**Table 1:** Protocols for the induction of hepatic differentiation with chromatin-remodeling agents.

Step	Time	Condition					
		G1	G2	G3	G4	G5	G6
Pretreatment	24 h	5-Aza-dC	5-Aza-dC	5-Aza-dC			
Conditioning	48 h						
Induction	7 d						
Differentiation	7 d	TSA			TSA		
Maturation	7 d	TSA	TSA		TSA	TSA	
